# The effect of perceived stress on depression in stroke: the chain mediating role of self-acceptance and self-perceived burden

**DOI:** 10.3389/fpsyg.2025.1694875

**Published:** 2026-01-09

**Authors:** Bing Li, Chundi Peng, Chunyan Sui, Weiye Chen, Xin Miao, Ye Zhou, Zhengxue Qiao

**Affiliations:** 1 Department of Mental Psychology, The Second Affiliated Hospital of Harbin Medical University, Harbin, Heilongjiang, China; 2 School of Mental Health, Wenzhou Medical University, Wenzhou, China; 3 Psychology and Health Management Center, Harbin Medical University, Harbin, Heilongjiang, China; 4 Harbin Medical University, Harbin, Heilongjiang, China

**Keywords:** stroke, depression symptoms, perceived stress, self-acceptance, self-perceived burden

## Abstract

**Background:**

This study aims to investigate the mechanism through which perceived stress affects depression symptoms by assessing the current status of depression symptoms and psychological characteristics in stroke patients.

**Methods:**

A total of 222 stroke patients were enrolled through convenience sampling at a tertiary general hospital in Harbin City during 2023–2024. Measurement tools included the patient health questionnaire-9, Chinese version of perceived stress scale, self-perceived burden scale and self-acceptance questionnaire. SPSS software was used for descriptive statistical analysis, *t*-test, analysis of variance, correlation analysis, multiple stepwise regression analysis and mediation effect test.

**Results:**

In this study, 67.12% of stroke patients had depression symptoms. The results of correlation analysis showed that perceived stress was significantly positively correlated with self-perceived burden and depression symptoms (*r* = 0.212–0.241, *p* < 0.01), and significantly negatively correlated with self-acceptance (*r* = −0.320, *p* < 0.01). Self-perceived burden was positively correlated with depression symptoms (*r* = 0.348, *p* < 0.01), and negatively correlated with self-acceptance (*r* = −0.255, *p* < 0.01). Self-acceptance was negatively correlated with depression symptoms (*r* = −0.304, *p* < 0.01). Mediation effect analyses showed that self-acceptance mediated significantly (95% CI [0.025, 0.117]); self-perceived burden mediated significantly (95% CI [0.003, 0.098]); and self-acceptance and self-perceived burden mediated significantly the chain of mediation in the relationship between perceived stress and depression symptoms (95% CI [0.005, 0.035]).

**Conclusion:**

Patients with stroke have a relatively high level of depression symptoms. Through a chain path of reducing perceived stress, enhancing self-acceptance, and alleviating perceived burden, the depression symptoms of stroke patients can be effectively alleviated.

## Highlights

- Findings from this research contributes to a deeper understanding of the pathogenesis and psychological mechanism of depression symptoms in stroke, and also provides strong theoretical support and practical guidance for clinical practice.
- This paper found that self-acceptance and self-perceived burden played a key mediating role in the influence of perceived stress on depression symptoms in stroke patients.
- The present research helps to improve the treatment effect, shorten the rehabilitation period of patients and improve their quality of life.

## Introduction

Post-stroke depression (PSD) is a typical psychological complication in stroke patients, and its clinical characteristics are persistent low mood and significant mood fluctuations (Moretti et al., 2015). Epidemiological studies have shown that the occurrence frequency of PSD at different time points after stroke presents dynamic distribution characteristics: the incidence of acute stage (within 1 month), subacute stage (1–6 months) and chronic stage (more than 6 months) are 33, 33 and 34%, respectively. Studies have shown that about one-third of stroke survivors will suffer from depression symptoms, which not only significantly delays the rehabilitation process of limb motor function, but also leads to decreased ability of daily living (Zhou et al., 2024), and increases the burden on families and society of patients (Medeiros et al., 2020). Therefore, in-depth discussion and scientific research on depression symptoms in stroke patients are urgent.

Perceived stress refers to the pressure intensity that individuals perceive subjectively when facing external stress events that exceed their coping ability (Van den Boogert et al., 2022). Previous studies have confirmed that there is a significant positive correlation between perceived stress and depressive symptoms, and it has a positive predictive effect on depressive symptoms in stroke patients (Engidaw et al., 2019; Guo et al., 2024). From the perspective of the mechanism of action, the perception of pressure will aggravate the mental tension and psychological burden of patients, and this continuous psychological stress state will indirectly promote the generation and development of depression symptoms through a series of physiological and psychological interactions (Muñoz-Peña et al., 2025). In addition, the pre-onset health status of stroke patients is also related to psychological factors to a certain extent. Patients are often accompanied by a history of chronic diseases such as hypertension and diabetes before the onset of stroke, and most of them lack systematic and standardized treatment and rational drug use guidance, which are easy to cause psychological pressure and then depression symptoms (Van Rijsbergen et al., 2019).

Self-acceptance is the emotional and attitudinal acceptance of one’s real self (Martin and Davies, 2008). Studies have confirmed a significant negative correlation between self-acceptance and depression symptoms (Sanghvi et al., 2023). When an individual’s self-acceptance is insufficient, it is easy to fall into depression symptoms, depression symptoms and other negative emotional states due to the gap between expectation and reality. In addition, relevant studies have further revealed the complex relationship between perceived stress, self-acceptance and postpartum depression (Jingwen et al., 2018). When individuals face the pressure brought by the epidemic, their choice of coping strategies is largely influenced by the level of self-acceptance. Therefore, it can be speculated that self-acceptance may play a mediating role between perceived stress and depression symptoms.

Self-perceived burden refers to the negative emotional experiences such as self-blame and reduced sense of self-identity that are often triggered when an individual suffers a major setback due to limited physical activity (Saji et al., 2023). These emotional responses not only constitute the core content of self-perceived burden, but also show a close correlation with mental health status in specific disease groups. Studies have shown that self-perceived burden can predict the occurrence and development of post-stroke depressive symptoms. Especially in young and middle-aged stroke patients, the level of self-perceived burden is significantly positively correlated with the severity of depressive symptoms (Ren et al., 2016). According to Lazarus and Folkman’s theory of stress-cognitive interaction, patients may view their own condition as a serious stressor and conduct negative cognitive evaluations of it, thereby intensifying their self-perceived burden (Lazarus, 1976). Based on the above findings, it can be inferred that self-perceived burden plays a mediating role between perceived stress and depression symptoms.

Within the framework of cognitive behavioral theory (Ahrens et al., 2023), self-perceived burden can be conceptualized as a specific emotional and cognitive syndrome that individuals develop due to low self-acceptance. When an individual falls ill, they attribute the stress or fatigue of their caregivers to themselves. This self-critical cognitive model continuously triggers patients’ feelings of guilt and shame, thereby constituting the experience of self-perceived burden. Previous studies have found that stroke patients with low self-acceptance are more likely to overestimate the economic and care burden they bring to their families (Huang et al., 2025; Jiru-Hillmann et al., 2022). This sequential association confirms the relationship between self-acceptance and self-perceived burden.

Although existing literature has extensively confirmed the association between perceived stress and depressive symptoms, the combined effects of two mediating variables—self-acceptance and self-perceived burden—have not been systematically explored in stroke patients. Previous studies have mostly focused on the basic mechanisms of depressive symptoms, with insufficient in-depth analysis of their specific pathways. Therefore, based on the cognitive interaction theory of stress, this study adopts a serial mediation model to simultaneously examine the mediating roles of self-acceptance and self-perceived burden in the relationship between perceived stress and depressive symptoms among stroke patients for the first time. By revealing this complex mediating pathway, this study aims to provide a more refined theoretical basis and practical suggestions for psychological interventions in stroke patients.

## Methods

### Participants and procedures

This study adopted convenient sampling method from 2023 to 2024, selected 250 stroke patients in a top-three hospital in Harbin city as samples, and finally collected 222 valid questionnaires. Participants were required to meet the following inclusion criteria: stroke patients diagnosed by CT/MRI, aged≥18 years, undergoing in-hospital treatment/rehabilitation, conscious and capable of basic language communication, and voluntarily signed informed consent. Excluding those who have speech difficulties or have recently experienced major life changes (such as divorce, widowhood). This paper was approved by the Ethics Committee of Harbin Medical University, and all participants completed the questionnaire with informed consent.

### Measures

The general data questionnaire was compiled by the researchers, which mainly investigated the gender of the patients, occupational category, the monthly income of the family per capita, and the payment method of personal medical expenses.

The Patient Health Questionnaire-9 (PHQ-9) covers 9 questions (Kroenke et al., 2001). The scoring standard is Likert 4-point scoring method, the higher the score, the more serious the depression symptoms status of the individual. This scale is a validated tool for assessing depressive symptomatology, though it is not a diagnostic instrument for clinical depressive disorder. The Chinese version has been proven to have good reliability and validity in the Chinese stroke patient population (Strong et al., 2021), with a Cronbach’s α coefficient of 0.912 in the present work.

The Chinese version of Perceived Stress Scale (CPSS-14) includes two dimensions of tension and sense of loss of control, and contains 14 assessment items. The scoring standard is Likert 5-point scoring method, and the score of the scale directly reflects the degree of pressure felt by individuals at the subjective level (Folkman et al., 1986). The Chinese version has been proven to have good reliability and validity in the Chinese stroke patient population (Li et al., 2022), with a Cronbach’s α coefficient of 0.851 in the present work.

The Self-Perceived Burden Scale (SPBS) divides the sense of burden into three dimensions: emotional burden, financial burden, and physical burden, and is an assessment instrument consisting of 10 items (McPherson et al., 2007). The scoring standard was Likert 5-point scoring method, the total score range of the whole scale was 10–50 points, and the total score was less than 20 points regardless of the influence of self-perceived burden. Mild score is 20–29; Moderate score was 30–39. A total score greater than 40 is classified as severe. The Chinese version has been proven to have good reliability and validity in the Chinese stroke patient population (Wei et al., 2020), with a Cronbach’s α coefficient of 0.916 in the present work.

The Self-Acceptance Questionnaire (SAQ), designed in 1999, includes 16 items in two dimensions: self-acceptance and self-evaluation (Cong and Gao, 1999). The Likert4 scale is used, with 1 to 4 representing “very opposite” to “very same” respectively. Among them, the reverse score is 1, 4, 7, 8, 11, 13, 14, and 16. The dimensions of self-acceptance and self-evaluation range from 8 to 32 points, and the total score ranges from 16 to 64 points. A high score means a higher level of self-acceptance. The Chinese version has been proven to have good reliability and validity in the Chinese patient population (Lu et al., 2022), with a Cronbach’s α coefficient of 0.808 in the present work.

### Statistical methods

Epidata 3.1 software was used for data entry, and SPSS 27.0 statistical software for statistical analysis, with a significance level of *p* < 0.05 to determine statistically significant differences. Prior to conducting parametric tests (e.g., *t*-tests and ANOVA), the assumptions of normality and homogeneity of variances were examined. The Shapiro–Wilk test indicated that all continuous variables were normally distributed (*p* > 0.05), and Levene’s test confirmed that the assumption of homogeneity of variances was met (*p* > 0.05). Therefore, the use of parametric tests was deemed appropriate. Descriptive statistical analysis, *t*-test, analysis of variance, correlation analysis, multiple stepwise regression analysis and mediation effect test were used to analyze the data. Among them, *t*-test and analysis of variance are used to compare the differences of the study variables among different groups, while correlation analysis is used to explore the pairwise relationships between the variables, and multiple stepwise regression analysis was used to explore the specific effects of multiple independent variables on depression symptoms (as dependent variables). To verify the mediating effect Model in the theoretical hypothesis, the PROCESS macro (Model 6) developed by Hayes was adopted to test the chain mediating model. This model can effectively test path models composed of multiple mediating variables and is a commonly used and mature method for analyzing complex mediating mechanisms at present.

## Results

### Demographics

In this study, there were 129 men and 93 women; 86.0% of the patients were married, 50.9% lived in the county or rural areas, 32.4% of the patients had primary school education or less, and only 15.3% of the patients were employed. In addition, there were statistically significant differences in the depression scores of stroke patients in whether there is a relapse after the disease has been controlled, the impact of stroke on daily life, sequelae of stroke (see Table 1 for details).

**Table 1:** General demographic data of stroke patients (*n* = 222).

Variable	Group	Number of people	Constituent ratio%	*M* ± SD	*t/F*	*P*
Gender	Male	129	58.2%	7.71 ± 5.47	2.044	0.154
	Female	93	41.8%	7.67 ± 6.16		
Marital status	Married	191	86.0%	7.75 ± 5.75	0.243	0.622
	Single (divorced, widowed, unmarried)	31	14.0%	7.35 ± 5.87		
Place of Residence	City	109	49.1%	7.85 ± 6.24	0.112	0.895
	County seat or town	64	28.8%	7.66 ± 4.77		
	Rural	49	22.1%	7.39 ± 5.91		
Educational level	Never attended school	14	6.3%	5.14 ± 5.23	1.177	0.322
	Primary school or below	58	26.1%	8.33 ± 5.27		
	Junior high	72	32.4%	7.18 ± 5.25		
	High school or technical secondary	43	19.4%	8.40 ± 6.67		
	College degree or above	35	15.8%	7.86 ± 5.76		
Occupation	Incumbent	34	15.3%	7.50 ± 5.58	2.310	0.077
	Retired	87	39.2%	8.54 ± 6.15		
	Laid-off	20	9.0%	9.20 ± 5.34		
	Freelance, other	81	36.5%	6.49 ± 5.33		
Per capita monthly household income	<1,000 yuan	21	9.5%	7.10 ± 5.43	0.933	0.426
	1,000–3,000 yuan	89	40.1%	8.04 ± 4.97		
	3,000–6,000 yuan	73	32.9%	8.11 ± 6.48		
	More than 6,000 yuan	39	17.5%	6.44 ± 6.15		
Payment of medical expenses	Full public health care	6	2.7%	5.83 ± 6.08	1.028	0.394
	Medical insurance	122	55.0%	7.80 ± 5.95		
	Part of the public fund	20	9.0%	9.80 ± 5.93		
	Rural cooperative medical care	70	31.5%	7.10 ± 5.35		
	Full self-funding	4	1.8%	7.25 ± 4.99		
Time since onset of stroke	Within 1 year	115	51.8%	7.03 ± 5.31	2.390	0.070
	1–3 years	56	25.2%	9.39 ± 5.98		
	3–5 years	32	14.4%	6.91 ± 6.23		
	More than 5 years	19	8.6%	8.00 ± 6.29		
Whether there is a relapse after the disease has been controlled	Yes	92	41.4%	8.03 ± 6.41	8.335	**0.004**
	No	130	58.6%	7.45 ± 5.26		
The impact of stroke on daily life	Severe	81	36.5%	9.69 ± 5.92	12.807	**<0.001**
	General	102	45.9%	7.38 ± 5.44		
	Less or no	39	17.6%	4.36 ± 4.47		
Whether to understand the relevant knowledge of stroke rehabilitation	Very understand	20	9.0%	7.35 ± 4.87	0.126	0.945
	Understand	67	30.2%	8.03 ± 6.38		
	General	97	43.7%	7.53 ± 5.75		
	Do not understand	38	17.1%	7.71 ± 5.19		
Stroke type	Ischemic stroke	170	76.6%	7.10 ± 5.57	0.031	0.861
	Hemorrhagic stroke	52	23.4%	9.63 ± 5.99		
Sequelae of stroke	None	56	25.2%	5.05 ± 4.86	6.927	**<0.001**
	Disability of physical activity	124	55.9%	8.31 ± 5.45		
	Swallowing disorders	11	5.0%	8.64 ± 4.54		
	Cognitive and mental disorders	8	3.6%	15.75 ± 6.02		
	Language disorders	15	6.7%	8.53 ± 7.48		
	Other	8	3.6%	5.63 ± 4.00		
Previous history: Diabetes	Yes	96	43.2%	8.31 ± 5.68	0.016	0.899
	No	126	56.8%	7.22 ± 5.78		
Previous medical history: Hypertension	Yes	154	69.4%	8.22 ± 5.90	2.046	0.154
	No	68	30.6%	6.50 ± 5.26		
Previous history: Hyperlipidemia	Yes	104	46.8%	8.48 ± 6.07	3.494	0.063
	No	118	53.2%	7.00 ± 5.39		
Previous history: Coronary heart disease	Yes	53	23.9%	9.66 ± 6.35	2.325	0.129
	No	169	76.1%	7.08 ± 5.43		
Previous history: Family history of stroke	Yes	58	26.1%	8.67 ± 5.77	0.163	0.687
	No	164	73.9%	7.35 ± 5.73		
Previous history: Smoking	Yes	102	45.9%	7.67 ± 5.40	1.692	0.195
	No	120	54.1%	7.72 ± 6.07		
Previous history: Alcohol consumption	Yes	81	36.5%	7.10 ± 5.49	0.432	0.512
	No	141	63.5%	8.04 ± 5.89		

Bolded values indicate *p* < 0.05.

### Depression status of stroke patients

The score of depression symptoms in this study was (7.69 ± 5.75); Among the 222 cerebral patients, 149 were depressed (67.12%). Among them, 72 were mild depression symptoms (32.43%). Forty-seven were moderately depressed (21.17%); 23 cases were moderate to severe depression symptoms (10.36%). Seven were severe depression symptoms, with an incidence of 3.15%, as shown in Table 2.

**Table 2:** Depression symptoms degree of stroke (*n* = 222).

Degree of depression symptoms	*n* (%)
None	73(32.89)
Mild depression symptoms	72(32.43)
Moderate depression symptoms	47(21.17)
Moderate to severe depression symptoms	23(10.36)
Severe depression symptoms	7(3.15)

### Correlation analysis among depression symptoms, perceived stress, self-acceptance and self-perceived burden

The results of correlation analysis showed that perceived stress was significantly positively correlated with self-perceived burden and depression symptoms (*r* = 0.212–0.241, *p* < 0.01). Perceived stress significantly negatively correlated with self-acceptance (*r* = −0.320, *p* < 0.01), suggesting that higher stress levels may substantially undermine a patient’s self-acceptance. Self-perceived burden was positively correlated with depression symptoms (*r* = 0.348, *p* < 0.01), and negatively correlated with self-acceptance (*r* = −0.255, *p* < 0.01). Self-acceptance was negatively correlated with depression symptoms (*r* = −0.304, *p* < 0.01). The overall pattern of results validates the clinical relevance of our theoretical model, suggesting that an integrated approach addressing stress, self-acceptance, and perceived burden may be more effective than focusing on any single factor alone (see Table 3 for details).

**Table 3:** Correlation analysis among study variables.

Item	1	2	3	4
Depression symptoms	1			
Perceived stress	0.212^**^	1		
Self-perceived burden	0.348^**^	0.241^**^	1	
Self-acceptance	−0.304^**^	−0.320^**^	−0.255^**^	1

**p < 0.01.

### Regression analysis

We used multiple stepwise regression analysis to explore the specific effects of multiple independent variables on depression symptoms (as the dependent variable). To control for potential confounding effects, we included key clinical variables (whether there is a relapse after the disease has been controlled, the impact of stroke on daily life, sequelae of stroke) as covariates in the regression model. As shown in Table 4, the perceived stress of stroke patients had a significant negative impact on self-acceptance (*β* = −0.34, *t* = −5.21**), and the perceived stress of stroke patients had a significant positive impact on self-perceived burden (*β* = 0.17, *t* = 2.52*). Stroke patients’ self-acceptance had a significant negative impact on their perceived burden (*β* = −0.20, *t* = −2.94**), in addition, stroke patients’ perceived stress had a significant positive impact on depression symptoms (*β* = 0.14, *t* = 2.33*), and self-acceptance had a significant negative impact on depression symptoms (*β* = −0.18, *t* = −3.03**), self-perceived burden had a significant positive effect on depression symptoms (*β* = 0.24, *t* = 3.96**).

**Table 4:** Test of mediating effect.

Regression equation		Overall fit index			Significance of regression coefficient	95% confidence interval
Outcome variable	Predictive variable	*R^2^*	*F*	*β*	*t*	95%CI
Self-acceptance	Perceived stress	0.11	6.96	−0.34	−5.21	[−0.25, −0.11]
Self-perceived burden	Perceived stress	0.11	5.53	0.17	2.52	[0.03, 0.26]
	Self-acceptance			−0.20	−2.94	[−0.52, −0.10]
Depression symptoms	Perceived stress	0.28	14.35	0.14	2.33	[0.01, 0.16]
	Self-acceptance			−0.18	−3.03	[−0.34, −0.07]
	Self-perceived burden			0.24	3.96	[0.08, 0.25]

### Test of chain mediation effect

**Figure 1:**
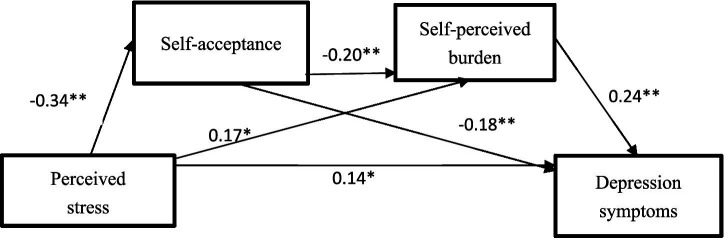
The chain mediation model of perceived stress → depression symptoms. **p < 0.01, *p < 0.05.

The in-depth analysis results of Bootstrap test clearly pointed out that the indirect effects of the three paths showed significance, specifically, the 95% confidence interval of these effects did not contain the value 0. As shown in Table 5, mediation effect analysis showed that self-acceptance played a significant mediating role in the relationship between perceived stress and depression symptoms (95%CI [0.025, 0.117]). The mediating effect of self-perceived burden on the relationship between perceived stress and depression symptoms was significant (95%CI [0.003, 0.098]). Self-acceptance and self-perceived burden had significant chain mediating effects on the relationship between perceived stress and depression symptoms (95%CI [0.005, 0.035]), and the indirect effect sizes of the three were 40.00, 26.87, and 10.63%, respectively. The model is shown in Figure 1.

**Table 5:** Bootstrap analysis of mediating effect significance.

Path	Estimate	BootSE	95%CI	Indirect effect size
X → M1 → Y	0.064	0.023	[0.025, 0.117]	40.00%
X → M2 → Y	0.043	0.025	[0.003, 0.098]	26.87%
X → M1 → M2 → Y	0.017	0.007	[0.005, 0.035]	10.63%
Total mediating effect	0.124	0.034	[0.064, 0.195]	77.50%
Direct effect	0.087	0.037	[0.014, 0.161]	54.37%
Total effect	0.160	0.037	[0.087, 0.233]	100%

X: Perceived stress; Y: Depression symptoms; M1: Self-acceptance; M2: Self-perceived burden.

## Discussion

The depression symptoms score of stroke patients was (7.69 ± 5.75), and the incidence of depressive symptoms stood at 67.12%. Compared with previous studies in China, the incidence of depressive symptoms in stroke patients (Chau et al., 2021), the results of this study showed a significant upward trend. After in-depth analysis, there may be the following reasons: The age structure of stroke patients is gradually becoming younger. Previous studies have clearly confirmed that young age is an important risk factor for post-stroke depression symptoms (Gurková et al., 2023). The younger age of onset means that more relatively young patients are facing the physical and mental challenges brought about by stroke, and patients may fall into anxiety due to fear that the disease will affect their work, and then develop depression symptoms. The group of stroke patients included in this study has the characteristics of long course and complex disease. Such patients often face higher disease uncertainty and prognostic concerns. The long course of treatment, the recurrence of the condition and the possible complications increase the psychological burden of the patient, which increases the risk of depressive symptoms. Finally, the differences among the study samples, the differences in the assessment time of depressive symptoms and the different assessment tools used may also affect the statistical results of the incidence of depression symptoms to some extent, resulting in the bias between previous studies. Factors such as the characteristics of different samples, the choice of evaluation time, and the sensitivity of evaluation tools may lead to differences in statistical results.

The pairwise correlation coefficients (*r* = 0.2–0.3) among the variables in the study were relatively low, indicating a limited direct association. However, this precisely supports the hypothesis that the impact of perceived stress on depression in stroke patients is mainly not a direct effect but is achieved through a chain mediating path composed of self-acceptance and self-perceived burden. Therefore, in clinical practice, focusing solely on the direct association between “stress and depression” may not be sufficient to reveal its intrinsic psychological mechanism. The chain mediation provides a targeted target for psychological intervention, blocking the transformation from stress to depression by enhancing self-acceptance and reducing the sense of burden, thereby providing a new theoretical basis and practical direction for the prevention and control of post-stroke depression.

This research identified the internal relationship between perceived stress and depression symptoms, that is, perceived stress can positively predict depression symptoms. Specifically, the perceived level of stress in stroke patients showed a significant positive correlation with the severity of depressive symptoms, and the more intense the perceived stress was, the more obvious the depressive tendency was. This finding is consistent with previous studies, which jointly revealed the psychological mechanism that perceived stress may increase the probability of depression symptoms by weakening patients’ sense of self-identity (Laures-Gore and Defife, 2013). It provides an important theoretical basis for understanding the complex relationship between stress and depression symptoms. When stroke patients go through the acute onset period and the long rehabilitation process, they often need to bear the pressure from multiple dimensions such as physiology, psychology and society. From the physiological point of view, the physical function limitation caused by the disease itself has brought great inconvenience and pain to patients. At the psychological level, factors such as changing family roles, lack of social support and potential stigma further increase the psychological burden of patients. In terms of cognition, most stroke patients hold a pessimistic attitude toward the prospects of rehabilitation, believing that the possibility of functional recovery is very small. This negative expectation not only reduces their treatment compliance, but also contributes to depression symptoms to a certain extent (Van Mierlo et al., 2015). In addition, the perception of stress can also promote the formation of patients’ negative cognitive bias toward the disease, that is, patients pay too much attention to the negative information of the disease, and ignore the positive factors such as the positive progress in the treatment process and the care given by the family. This cognitive imbalance further aggravates patients’ depressive mood (Zhang et al., 2015). At the same time, high stress perception also has a negative impact on patients’ emotional regulation ability. It impairs the patient’s ability to adopt effective coping strategies in the face of negative emotions, making it difficult for the patient to cope with depressive symptoms, thus exacerbating the manifestation of depression symptoms.

Self-acceptance plays a partial mediating role in the relationship between perceived stress and depression symptoms, and can effectively alleviate the negative effects of perceived stress on individual depression symptoms. For stroke patients, they often need to withstand multiple pressures from the external environment such as the disease itself and the change of social roles. Chronically exposed to such stressful situations, patients may find it difficult to accept their status quo (Ma et al., 2021). When the degree of self-acceptance is insufficient, patients will show more negative emotions and attitudes in the interaction with family members and medical staff. These manifestations not only tend to destroy their interpersonal relationships, but also lead to a decrease in social support. The lack of social support will further aggravate the helplessness of patients, and even induce more serious depression symptoms. However, previous studies have fully confirmed that in the face of psychological distress, self-acceptance, as a positive psychological trait, plays a crucial role in emotional regulation. Based on the theoretical framework of the stress process model, this study believes that when patients encounter stressful events, such as their own diseases, their original physical and mental balance will be challenged, and then trigger internal psychological protection mechanisms such as self-acceptance to cope with the threat of stressful stimuli. In the process of overall nursing and rehabilitation of stroke patients, it is important to promote self-acceptance. This not only helps patients face the disease situation more calmly, but also plays a positive role in preventing and alleviating depressive symptoms. This is because self-acceptance can prompt patients to respond to stressful situations in a more positive and constructive way (Luo et al., 2020; Tong et al., 2021).

The results show that self-perceived burden is a key mediating variable, which plays a mediating role in the relationship between perceived stress and depressive. Self-perceived burden refers to the negative emotional experience that an individual may bring psychological or actual burden to others due to his own health status and dependence on care services. The score directly reflects the burden felt by the caretakers. From the perspective of equity theory, individuals tend to maintain a balance between receiving and giving help in interpersonal interaction (Mcpherson et al., 2007). However, due to the change in the role of stroke patients after illness, they provide significantly less help and support to caregivers and rely more on external support and care (Maggio et al., 2024). This balance is broken, resulting in emotional inequality, that is, emotional burden. In addition, chronic diseases, especially the long-term rehabilitation process of stroke, not only impose a heavy financial burden on patients, but also further aggravate the physical burden of patients due to continued impairment of physical functions (Sun et al., 2024; He et al., 2023). These factors are interwoven and jointly promote the development of depression symptoms. Based on the above analysis, in the treatment and care system for stroke patients, medical personnel should give full play to their initiative and empathy, regularly conduct in-depth communication with patients, and fully understand patients’ psychological state, physical feelings and potential concerns. Family members should also actively participate in the patient’s recovery process and provide emotional support for the patient. The companionship and care of family members can enhance the psychological resilience of patients, reduce their self-felt burden, and then have a positive impact on improving the depression symptoms of patients.

Self-acceptance and self-perceived burden showed a significant chain mediating effect on the influence of perceived stress on depression symptoms. Specifically, when an individual’s perceived level of stress is elevated, their self-acceptance is significantly reduced. This decrease in self-acceptance leads to an even greater burden of self-feeling. Once the self-perceived burden is formed, individuals will be more inclined to adopt catastrophic thinking and self-blame coping methods when facing stressful events. However, such coping methods not only fail to effectively solve the problem, but also do not help the recovery of the illness, and can contribute to the worsening of depression symptoms. In contrast, high levels of self-acceptance can enable individuals to show greater mental resilience and flexibility in the face of stress. Individuals with this trait can fully and deeply experience and accept their own emotional changes, thus effectively avoiding the generation of negative cognition (Simmons, 2007). For this particular group of stroke patients, they bear a heavy burden of disease for a long time, often accompanied by a low level of self-acceptance. These two factors interweave and influence each other, significantly increasing the patient’s self-perceived burden, and then become an important factor inducing depression symptoms. This study highlights that there is a negative correlation between self-acceptance and self-perceived burden. Specifically, by improving the self-acceptance level of patients, it helps to improve their understanding and acceptance of the disease, and reduce the fear of the disease itself. On this basis, patients can more effectively manage the burden of self-perception, achieve emotional balance and stability, and finally have a positive and significant impact on relieving depression symptoms. Therefore, it can strengthen emotional communication, avoid excessive criticism and blame, and create a supportive family environment. Disseminate mental health knowledge through community publicity, media reports and school education. Establish community support networks, patient mutual aid organizations, and improve social security to reduce economic burdens. Psychological factors should be considered in the formulation of medical plans, and doctor-patient communication should be strengthened to improve treatment compliance.

### Limitations and strength

Our study has several limitations. First, the cross-sectional design precludes the establishment of definitive causal relationships among the variables, only revealing their associations at a single time point. This design also falls short of addressing the need for longitudinal or intervention-based research. Second, the limited sample size may constrain the external validity of the findings. Moreover, the exclusive reliance on self-reported measures for data collection introduces potential biases, such as social desirability bias and participants’ subjective cognition or recall biases. Additionally, the potential influence of cultural bias has not been sufficiently discussed in this study. Future research could consider expanding the sample size, adopting longitudinal or experimental designs, and incorporating diverse methods such as qualitative interviews to more comprehensively and deeply elucidate the relationships among variables.

## Conclusion

Overall, stroke patients had higher levels of depression symptoms. There were significant correlations among depression symptoms, perceived stress, self-acceptance and self-perceived burden. Self-acceptance and self-perceived burden play a chain mediating role between perceived stress and depression symptoms.

## Data Availability

The raw data supporting the conclusions of this article will be made available by the authors, without undue reservation.
